# Acupuncture for balance dysfunction in patients with stroke: A systematic review protocol

**DOI:** 10.1097/MD.0000000000011681

**Published:** 2018-08-03

**Authors:** Lei Xu, YouKang Dong, Min Wang, LiQiu Chen, ZeRong Zhang, DongSheng Su, Feng Zhang, Zhen Lei, WeiYong Xu, Kuete Kamtsop Christian Didier, YuanHao Du

**Affiliations:** a Department of Rehabilitation Medicine, The First Affiliated Hospital of Bengbu Medical College, Bengbu Medical College, Bengbu, Anhui; b Tianjin University of Traditional Chinese Medicine, Tianjin; c The First Affiliated Hospital of Yunnan University of Traditional Chinese Medicine, Kunming, Yunnan; d Department of Rehabilitation Medicine, The Second Affiliated Hospital of Bengbu Medical College, Bengbu Medical College; e Department of Rehabilitation Medicine, No 3 Hospital of Bengbu, Bengbu, Anhui; f Department of Rehabilitation Medicine, Municipal Hospital of Suzhou, Suzhou, Anhui, China.

**Keywords:** acupuncture, balance dysfunction, protocol, stroke, systematic review

## Abstract

**Background:**

Balance dysfunctions in stroke survivors are common and have significant impact on functional independence and rehabilitation. As a crucial technique of Traditional Chinese Medicine, acupuncture has been used widely for balance dysfunctions after stroke, although its effective evidence is not clear. Hence, we plan this systematic review protocol to evaluate the value of its efficacy and safety for balance dysfunctions after stroke.

**Methods:**

We will search the databases from the publishment to April 2018: Web of Science, PubMed, Medline, Cochrane Library, EBASE, WHO International Clinical Trials Registry Platform, Wanfang, Chinese Biomedical Literature Database, Chinese Scientific Journal Database (VIP), and China National Knowledge Infrastructure. The clinical efficacy will be accepted as the primary outcomes. RevMan V.5.3 software will be used to compute the data synthesis when a meta-analysis is allowed.

**Results:**

This systematic review and meta-analysis will provide a high-quality synthesis of current evidence of acupuncture for balance dysfunctions after stroke including clinical efficacy, balance ability, walking ability, and activity of daily life etcetera.

**Conclusion:**

This protocol will determine whether acupuncture is an effective and safety intervention for balance dysfunctions after stroke.

## 1 Introduction

Stroke is a major cause of disability and dependency, which brings huge detriment to individuals and heavy burden to families and societies.^[1]^ In China, stroke is also the main cause of death and long-term disability with the amount of annual stroke mortality of approximately 1.6 million.^[2]^ An epidemiological study suggested that the incidence of stroke was 116 to 229 of 100,000 person-years, leaving approximately 75% of individuals with motor dysfunction and 40% with severe disability in China.^[3]^

Balance function refers to the body's ability to maintain a stable posture and automatically adjust and maintain posture when exercising or being subjected to external forces.^[4]^ Balance dysfunctions in stroke survivors are common and have significant impact on functional independence and rehabilitation.^[5]^ The balance function is the coordination between the vestibular system, vision, and body movements to improve muscle tone and activate muscle tendon, and ligaments, bones, and joints make balanced movements to maintain posture. It is the premise of all physical activity, and is an important part of rehabilitation after stroke. It also seriously affects the patient's walking and daily life activities, increases the risk of falls, and brings great psychological and economic burden to patients and their families. Balance disability affects >80% of patients poststroke,^[6]^ with falls occurring in 40% to 70% of stroke survivors within the first year.^[7]^ Balance dysfunction after stroke is related to poorer mobility, and ability to perform activities of daily living, and falls.^[8]^ Patients with hemiplegia after stroke have weakened or lost control of motor function on the affected side of the trunk. The patients’ standing balance and walking ability are limited due to the damage of balance center in the nervous system.^[9]^ Therefore, a vital goal of rehabilitation is to regain adequate balance function to facilitate the walk safely.^[10]^

Acupuncture is an important part of Traditional Chinese Medicine (TCM) which has been practiced in China over 3000 years,^[11]^ and is traditionally considered as a treatment for various ailments associated with stroke.^[12]^ Acupuncture is widely used to improve motor, sensation, speech, and other neurological functions in patients with stroke.^[13]^ Scalp acupuncture has a good effect on restoring limb movement and balance disability.^[14]^ According to TCM, stroke is caused by Qi deficiency and blood stasis, wind, fire, phlegm, emotions, and fatigue, which led to obstruction of vessel in brain or breach of vessel.^[15]^ Acupuncture and moxibustion can dredge meridians, supplement Qi and improve blood circulation, regulate Qi and expel phlegm, consciousness-restoring resuscitation, and other effects.^[16]^ Modern research confirms that acupuncture points in the body can enhance the afferent impulses received by the receptors to the cerebral cortex, promote the formation of motor conduction pathways, and restore limb function.^[17]^ Acupuncture can improve the ischemic and hypoxic conditions of cells around the lesion, and can promote the establishment of collateral circulation and absorption of hematoma.^[18]^ Although there are abundant of clinical studies about acupuncture, many clinicians still worry about its therapeutic effect and safety. In this review, we aim to perform a systematic review to assess and appraise the effectiveness and safety of acupuncture for balance dysfunction in patients with stroke.

## 2 Methods

This systematic review protocol has been registered on PROSPERO under the number of CRD42018095800, and was performed in accordance with the Preferred Reporting Items for Systematic Reviews and Meta-analysis Protocol.^[19]^ This is a literature-based study, so ethical approval is unnecessary.

### 2.1 Selection criteria

#### 2.1.1 Types of studies

In the research, all randomized controlled trials will be included without restrictions on language and publication status.

#### 2.1.2 Types of patients

Trials must include adult participants with balance dysfunction after stroke. The patient must meet the diagnostic criteria established by the Academic Conference on Cerebrovascular Disease and be confirmed by computed tomography or magnetic resonance imaging. Between the ages of 40 and 75 years including men and women, the basic vital signs of the patient are stable, and the Berg Balance Scale is <20 points.

#### 2.1.3 Types of interventions

Different technique of acupuncture will be included in this review such as scalp acupuncture, electroacupuncture, abdominal acupuncture, and balance acupuncture. Any methods can be used without limitations on the treatment courses, retaining time of needles, and frequency. The control groups will be treated with sham acupuncture or other training therapy.

#### 2.1.4 Types of outcomes

Primary outcomes will include balance ability, walking function, motorfunction improvement, self-care ability, and daily living as well as the side effects of acupuncture. Secondary outcomes will include quality of life, mental health, and degree of satisfaction with the treatment.

### 2.2 Search methods for the identification of studies

Relevant data bases include Web of Science, PubMed, Medline, Springer, Cochrane Library, EBASE, WHO International Clinical Trials Registry Platform, Wanfang, Chinese Biomedical Literature Database, Chinese Scientific Journal Database (VIP), and China National Knowledge Infrastructure from the publishment to April, 2018. The search strategy is listed in Table 1, which includes all search terms, and other searches will be conducted based on these results. Literatures that do not meet the inclusion criteria will be excluded. Any disagreement should be settled by discussion.

**Table 1:**
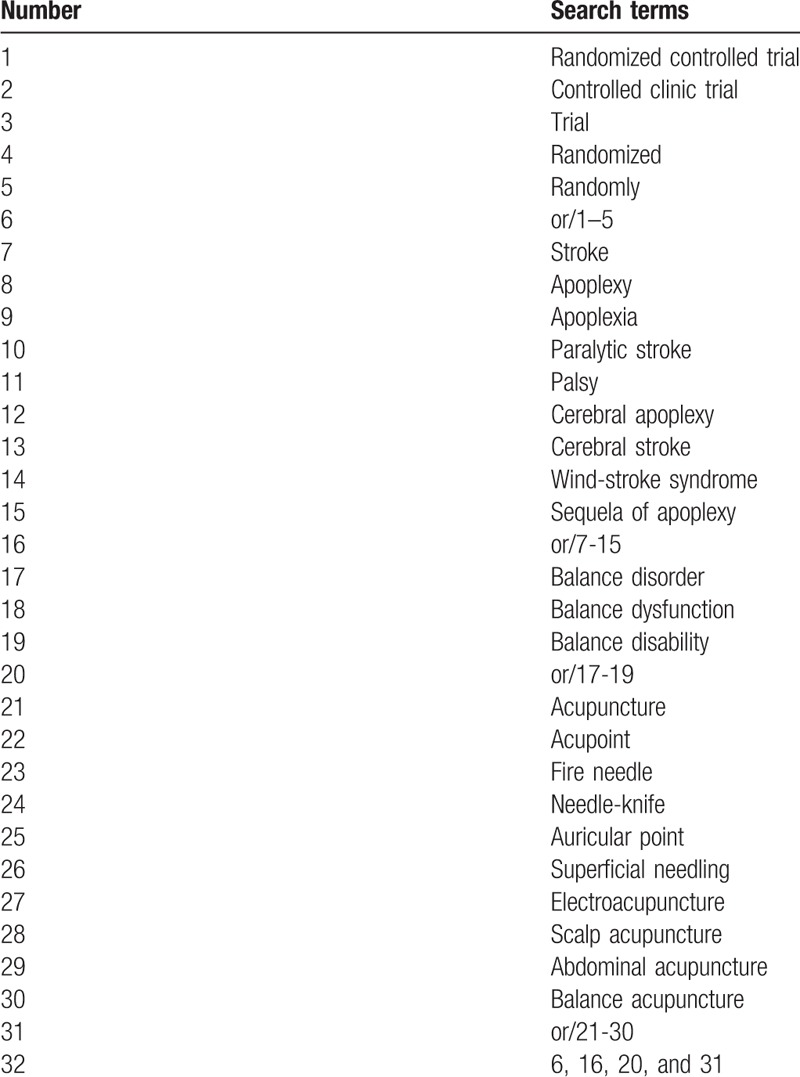
Web of science search strategy.

### 2.3 Data collection and analysis

#### 2.3.1 Selection of studies

**Figure 1:**
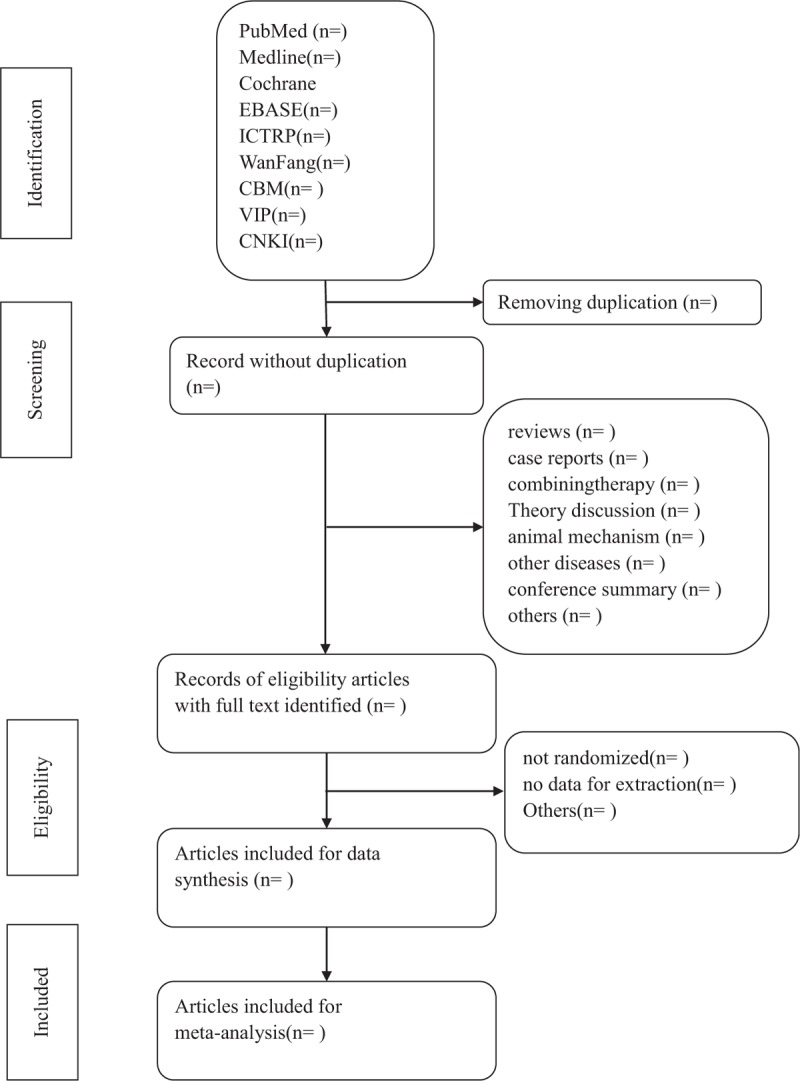
Flow diagram of studies identified.

Two researchers will search the keywords, abstracts, and titles of the eligible references independently. At last they will decide which trials can satisfy the inclusion criteria. If there are any disagreements, they will discuss and contact with the author to learn about the details of related researches. The study of screening flow diagram is summarized in Figure 1.

#### 2.3.2 Data extraction and management

Data from each selected article will be extracted by ZL and WX. We will extract information for publication time, allocation concealment, author, interventions, and so on. The third researcher named Feng Zhang will check data again. If necessary, we will consult the author of the article for further information. Any possible disagreements will be settled by arbiter and experts.

#### 2.3.3 Assessment of risk of bias in included studies

The risk of bias will be evaluated by YD and KKCD, respectively in accordance with the Cochrane collaboration's tool.^[20]^ We will assess the following 7 items: random sequence generation, allocation concealment, blinding, personnel and outcome, incomplete outcome data, selective reporting, and other issues. These items will be divided into 3 categories: low risk of bias, high risk of bias, and unclear risk of bias.

#### 2.3.4 Dealing with missing data

If we encounter missing data, we will try our best to contact researchers to obtain information by means of telephone, email, and so on. If missing data are not available, the analysis will be based on the available data.

#### 2.3.5 Heterogeneity assessment and data synthesis

The software RevMan5.3 from the Cochrane Collaboration will be used to carry out the meta-analysis. The dichotomous outcomes will be analyzed by the relative risk with 95% confidence interval (CI), and continuous outcomes will be evaluated by standard mean difference with 95% CI. The tests for heterogeneity in this study will be conducted by *I*^2^ statistics. If *I*^2^ < 50%, the fixed effect model will be used for pooled data. If *I*^2^ > 50%, the random effect model will be selected for pooled data in factors such as sex, age, acupuncture and acupoints, size of sample, and others might influence the consequences. In this case, subgroup analysis, meta regression analysis, and the sensitivity analysis based on the data will be taken into account and implemented to explore the causes of heterogeneity.

#### 2.3.6 Assessment of reporting bias

We will use Funnel plot to assess the reporting biases when the trials included in a meta-analysis over 9. Egger test will be performed to assess plots visually.

#### 2.3.7 Subgroup analysis

If we observe significant heterogeneity in the included studies, subgroups analysis will be performed according to age, sex, different interventions, controls, and outcome measures.

#### 2.3.8 Sensitivity analysis

If trials data are sufficient, the sensitivity analysis will be carried out to identify whether the conclusions are robust. We can evaluate it through sample size, the effect of missing data and so on.

#### 2.3.9 Ethics and dissemination

There is no requirement of ethical approval for the protocol. The protocol will be disseminated in a peer reviewed journal and presented at conference.

## 3 Discussion

Balance, a multifactorial phenomenon, is an ability to maintain upright and weight bearing posture within the base of support not leading to a fall.^[21]^ Balance dysfunction is one of the common complications of stroke. Patients with stroke with balance dysfunction are connected with sensory input, central system integration motion control, and other aspects. Problems in the cognitive system can also cause balance disorders.^[22]^ It seriously affects the patient's walking and daily life activities, increases the risk of falls, and brings great psychological and economic burden to patients and their families.^[23]^

Acupuncture has been used to treat stroke in many centuries; many doctors treat stroke patients to maximize the promotion of recovery.^[24]^ Acupuncture sends bioelectrical effects to the site of action, stimulating damaged nerves and muscles, causing passive contraction, and promoting muscle motor function and nerve regeneration. Acupuncture can improve the ischemic and hypoxic conditions of brain tissue, increase cerebral blood flow, promote the establishment of cerebral collateral circulation, and accelerate the repair of damage brain tissue.^[25]^ Acupuncture is an effective and safe intervention to treat balance dysfunction after stroke but lacks convincing and comprehensive evidence. Up to now, there is no relevant systematic review reported. Although there are still some limitations of the proposed systematic review especially different forms of acupuncture and acupoints adopted in the study, and difficulties in taking blind measures during acupuncture treatment might lead to significant heterogeneity, we will try to deal with such problems in a proper way.

Although there is no clear evidence from evidence-based medicine that acupuncture can improve the balance of patients with, some small-scale clinical trials have observed that acupuncture treatment can improve balance dysfunction after stroke, daily life skills, and walking skills. Patients can return to their families as soon as possible. It might be the first systematic review to analyze literature of acupuncture for balance dysfunction after stroke. We hope that this systematic review can provide a forceful proof of effectiveness and safety of acupuncture on balance dysfunction after stroke for clinicians, scientific researchers, and health policy conductors.

## Author contributions

**Conceptualization:** YuanHao Du.

**Investigation:** YouKang Dong, ZeRong Zhang.

**Methodology:** LiQiu Chen, Feng Zhang.

**Resources:** DongSheng Su, Zhen Lei, WeiYong Xu, Kuete Kamtsop Christian Didier.

**Supervision:** Min Wang.

**Writing – original draft:** Lei Xu.

**Writing – review and editing:** YuanHao Du.
